# Association between neuromuscular blocking agent use and outcomes among out-of-hospital cardiac arrest patients treated with extracorporeal cardiopulmonary resuscitation and target temperature management: A secondary analysis of the SAVE-J II study^☆^

**DOI:** 10.1016/j.resplu.2023.100476

**Published:** 2023-09-26

**Authors:** Masatoshi Uchida, Migaku Kikuchi, Yasuo Haruyama, Toru Takiguchi, Toru Hifumi, Akihiko Inoue, Tetsuya Sakamoto, Yasuhiro Kuroda, SAVE-J II study group

**Affiliations:** a Department of Emergency and Critical Care Medicine, Dokkyo Medical University, Tochigi, Japan; b Integrated Research Faculty for Advanced Medical Sciences, Dokkyo Medical University, Tochigi, Japan; c Department of Emergency and Critical Care Medicine, Nippon Medical School, Tokyo, Japan; d Department of Emergency and Critical Care Medicine, St. Luke’s International Hospital, Tokyo, Japan; e Department of Emergency and Critical Care Medicine, Hyogo Emergency Medical Center, Kobe, Japan; f Department of Emergency Medicine, Teikyo University School of Medicine, Tokyo, Japan; g Department of Emergency, Disaster and Critical Care Medicine, Kagawa University Hospital, Kagawa, Japan

**Keywords:** Cardiac arrest, Extracorporeal cardiopulmonary resuscitation, Targeted temperature management, Neuromuscular blocking agents

## Abstract

**Background:**

Neuromuscular blocking agents are used to control shivering in cardiac arrest patients treated with target temperature management. However, their effect on outcomes in patients treated with extracorporeal cardiopulmonary resuscitation is unclear.

**Methods:**

This study was a secondary analysis of the SAVE-J II study, a retrospective multicenter study of 2175 out-of-hospital cardiac arrest patients treated with extracorporeal cardiopulmonary resuscitation in Japan. We classified patients into those who received neuromuscular blocking agents and those who did not and compared in-hospital mortality and incidence rates of favorable neurological outcome and in-hospital pneumonia between the groups using multivariable regression models and stabilized inverse probability weighting with propensity scores.

**Results:**

Six hundred sixty patients from the SAVE-J II registry were analyzed. Neuromuscular blocking agents were used in 451 patients (68.3%). After adjusting for potential confounders, neuromuscular blocking agents use was not significantly associated with in-hospital mortality (aHR 0.88; 95% CI, 0.67–1.14), favorable neurological outcome (aOR 0.85; 95% CI, 0.60–1.11), or pneumonia (aOR 1.52; 95% CI, 0.85–2.71). The results for in-hospital mortality (aHR 0.89; 95% CI, 0.64–1.25), favorable neurological outcome (aOR 0.94; 95% CI, 0.59–1.48) and pneumonia (aOR 1.59; 95% CI, 0.74–3.41) were similar after weighting was performed.

**Conclusions:**

Although data on the rationale for using neuromuscular blocking agents were unavailable, their use was not significantly associated with outcomes in out-of-hospital cardiac arrest patients treated with extracorporeal cardiopulmonary resuscitation and targeted temperature management. Neuromuscular blocking agents should be used based on individual clinical indications.

## Introduction

Out-of-hospital cardiac arrest (OHCA) is a significant public health problem, with an estimated global incidence of 55 cases per 100,000 person-years.^1^ Several randomized controlled trials (RCTs) have shown the benefit of target temperature management (TTM) for patients with post cardiac arrest syndrome (PCAS).^2^, ^3^, ^4^ A recent RCT has reported that there was no significant difference in outcome between TTM at 33 °C and early treatment of fever.^5^ Consequently, current international guidelines recommend actively preventing fever.^6^ However, TTM is still considered to be a “good practice” for the management of patients with PCAS.^6^ Shivering is a common complication of TTM which makes it difficult to control temperature,^7^ increases oxygen consumption,^8^ and reduces brain oxygen supply.^9^ Control of shivering is essential for temperature management to prevent further brain damage.^7^

Extracorporeal cardiopulmonary resuscitation (ECPR) is cardiopulmonary resuscitation (CPR) combined with extracorporeal membrane oxygenation (ECMO) for refractory cardiac arrest.^10^ The use of ECPR is increasing worldwide^11^, ^12^ and a meta-analysis based on four RCTs^13^, ^14^, ^15^, ^16^ reported its benefit for neurological outcome.^17^ It is inferred that patients who were received ECPR have more severe brain damage and are more vulnerable to further brain injury because of their longer cardiac arrest duration and inflammation by ECMO use^18^ which is considered a contributory factor to secondary brain injury.^19^ Preventing further damage in these patients is essential.

Neuromuscular blocking agents (NMBAs) are frequently used to control shivering during TTM.^7^ Their use may improve outcomes in OHCA patients by suppressing shivering and optimizing TTM.^20^ Furthermore, NMBA have been reported to improve outcomes in acute respiratory distress syndrome (ARDS),^21^ thus they could potentially be applicable for treating severe hypoxemia or ARDS following OHCA. Although current guideline recommended to use NMBA only for severe shivering during TTM and to avoid routinely use,^22^ previous studies have provided conflicting results.^23^, ^24^, ^25^, ^26^, ^27^, ^28^, ^29^ In addition, few of the studies included patients who underwent ECPR. Therefore, the effects of NMBAs in ECPR patients have not been well studied. This study aimed to investigate the association between NMBAs and outcomes in OHCA patients treated with ECPR.

## Methods

### Study design

This retrospective observational study used data from the Study of Advanced life support for Ventricular fibrillation with Extracorporeal circulation in Japan (SAVE-J II) patient registry to conduct a secondary analysis.^30^ All patient data were de-identified prior to analysis. Approval was obtained from the institutional review board of Dokkyo Medical University. The requirement for patient consent was waived owing to the retrospective nature of the study.

### Participants

The SAVE-J II study enrolled 2157 consecutive patients over 18 years of age who presented to the emergency department with OHCA and underwent ECPR from 36 institutions in Japan between January 1, 2013, and December 31, 2018.^31^ Details of the ECPR procedure have been previously described.^30^ Our study included patients from the registry who received ECPR (i.e., ECMO insertion while in a state of cardiac arrest), underwent TTM and received sedatives within 24 hours of admission to the intensive care unit (ICU). These inclusion criteria were selected because all eligible patients in two previous studies that evaluated the association between the use of NMBAs and outcomes received sedatives.^25^, ^28^ Sedative agents are recommended in the acute phase of TTM and are considered standard of care for patients with PCAS undergoing TTM.^32^

Patients with non-cardiac conditions such as aortic dissection or aneurysm, hypothermia, primary cerebral disorder, infection, drug intoxication, trauma, suffocation, and drowning were excluded from analysis. We also excluded patients who achieved return of spontaneous circulation at hospital arrival or at ECMO initiation, those who were transferred from another hospital, and patients with incomplete or missing data regarding NMBAs, TTM, sedatives, or outcomes. Furthermore, to exclude patients who died or received a 'do not attempt resuscitation' (DNAR) order within 24 hours of ICU admission, we also excluded those who experienced either event by the second calendar day following their admission. This was because the registry only collected the date of these events, without exact time.

### Data collection

Patient demographic and clinical data were collected from the SAVE-J II study registry. Data details are presented in Supplementary Table 1.

### Exposure

The primary exposure in this study was any NMBA use, either as a bolus or continuous infusion, at ICU admission or within 24 hours of admission.

### Outcomes

The primary outcome was in-hospital mortality. Secondary outcomes were favorable neurological outcome at hospital discharge and pneumonia during hospitalization. Favorable neurological outcome was defined as Cerebral Performance Category score 1 or 2.^33^ Pneumonia was defined as the presence of infiltrates on chest radiography in conjunction with detection of bacteria in sputum cultures and at least one of the following: body temperature >38.0 °C, white blood cell count >12,000 cells/mL, and purulent sputum. Pneumonia was chosen as a secondary outcome for multivariable analysis because its definition was pre-established. Other outcomes like ventilator-associated pneumonia and atelectasis were also collected but were not included in the multivariable analysis, as they were solely subject to physician interpretation in the registry.

### Covariates

Adjustments were made for age, sex, witnessed cardiac arrest, bystander CPR, initial rhythm, location of cardiac arrest, and estimated cardiac arrest duration, and target temperature for TTM. The estimated cardiac arrest duration was defined as follows: for patients whose location of cardiac arrest was an ambulance, the time from cardiac arrest to the establishment of ECMO; for patients whose location of cardiac arrest was other than an ambulance, the time from calling an ambulance to the establishment of ECMO. The target temperature for TTM was determined based on the target temperature at the beginning of TTM. Patients with a target temperature ≤35 °C were classified as 'hypothermia'; those with target temperature ≥35.5 °C were classified as 'normothermia' according to a previous research.^34^ If the target temperature at the beginning of TTM was missing, the final target temperature after any adjustment was used. The adjusted covariates were selected based on previous studies^35^, ^36^, ^37^, ^38^, ^39^, ^40^, ^41^ and their clinical importance.

### Statistical analysis

Patients were grouped according to NMBA use (NMBA and no NMBA groups). Continuous variables are presented as medians with interquartile range (IQR) and were compared using the Mann–Whitney U test. Categorical variables are presented as numbers with percentage and were compared using the chi-square or Fisher's exact test as appropriate.

A multivariable Cox shared frailty model was constructed to investigate the association between NMBA use and hospital mortality while adjusting for covariates as described above. A Cox shared frailty model is a survival analysis model that accounts for unobserved heterogeneity among individuals in the same cluster.^42^ Multivariable logistic regression was performed with generalized estimating equation (GEE) modeling to investigate the association between NMBA use and secondary outcomes while accounting for patient clustering within hospitals. The adjusted covariates are described above.

Propensity score analysis was also conducted as sensitivity analysis to reduce potential confounders between NMBA use and outcomes. To calculate propensity scores for NMBA use, multivariable logistic regression to adjust for potential confounders (age, sex, witnessed cardiac arrest, bystander CPR, initial rhythm, location of cardiac arrest, estimated cardiac arrest duration, and target temperature for TTM) was performed. The c-statistic was used to assess the model's discriminative ability. Stabilized inverse probability weighting (sIPW) was used to estimate the average treatment effect.^43^ For patients who received NMBs weighted by the inverse of PS (1/PS) and those who did not receive NMBs weighted by the inverse of 1 minus their PS (1/(1-PS)). To prevent undue influence of extreme weights assigned to patients with a low probability of receiving NMBs, stabilized IPWs were created by multiplying the marginal probability of receiving NMBs by the IPW. Less than 10% of an absolute standardized mean difference for the covariates indicated a good covariate balance.^44^ Weighted Cox proportional hazards models were used to calculate adjusted hazard ratios (HRs) to evaluate the association between NMBA use and hospital mortality. Weighted logistic regression models were used to calculate adjusted odds ratios (ORs) to evaluate the association between NMBA use and secondary outcomes, both weighted by the stabilized IPW described above. For the weighted analyses, the sandwich variance estimator was used to calculate confidence intervals (CIs) and p-values.

Subgroup analyses were conducted using an interaction term between NMBA use and subgroups to evaluate potential effect modifiers. Subgroups were chosen based on a previous study,^34^ the potential for a lower targeted temperature to cause more shivering, and the possibility that patients with longer cardiac arrest duration may have a more severe brain injury and benefit more from NMBA use in preventing secondary brain injury. The subgroup categories were age (<65 vs. ≥65 years), target temperature for TTM (≤35 °C vs. >35 °C), and estimated cardiac arrest duration (≤50 minutes vs. >50 minutes). The interaction was tested using the Wald test.

All tests were two-tailed. *p* < 0.05 was considered significant. Statistical analyses were performed using R software version 4.2.0 (The R Foundation for Statistical Computing, Vienna, Austria).

## Results

### Patient characteristics

**Fig. 1:**
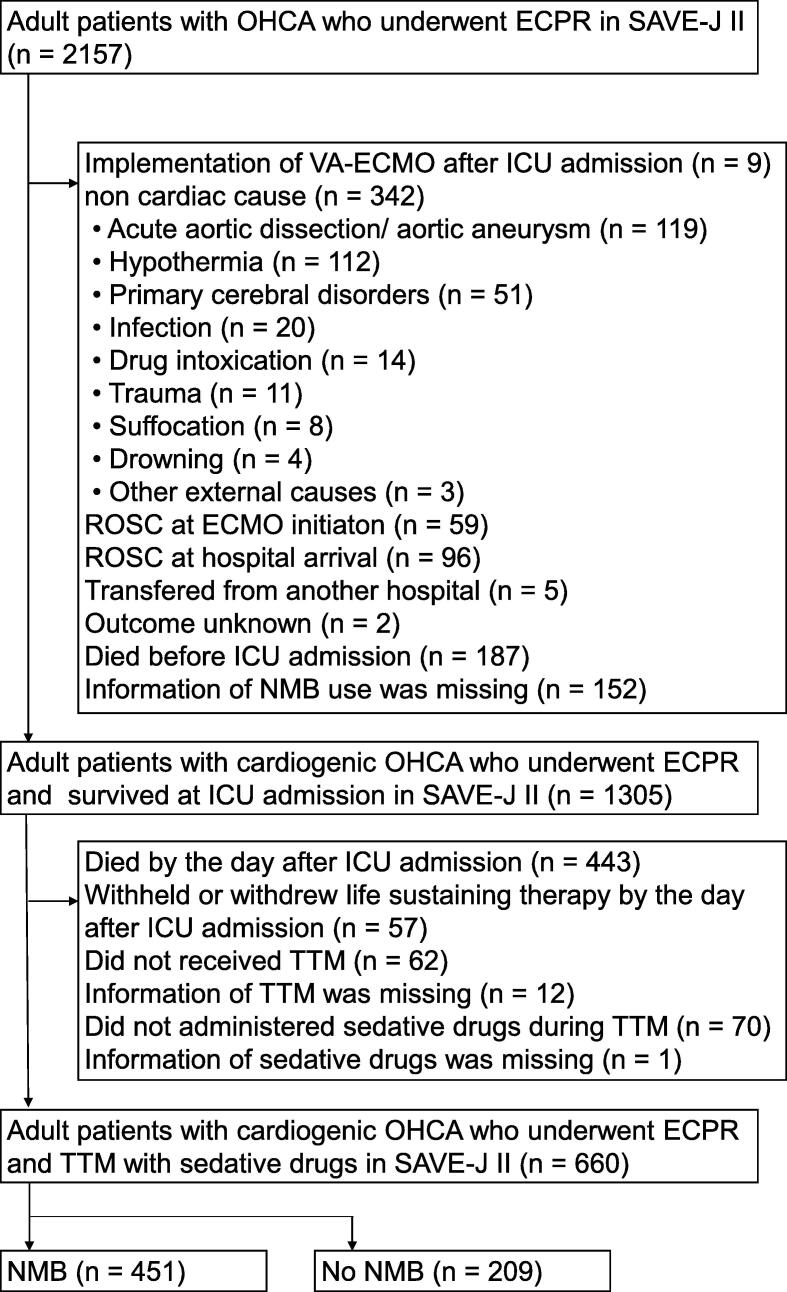
Study flow chart.

Among the 2157 patients in the SAVE-J II registry, 660 met the criteria described above and were included for analysis. NMBAs were administered to 451 patients (the NMBA group). A study flowchart is shown in Fig. 1. Patient characteristics are summarized in Table 1. Patients in the NMBA group were significantly younger (median age, 58 vs. 63 years; *p* < 0.001), more likely to be male (87.4% vs. 80.4%; *p* = 0.019), and had a shorter estimated cardiac arrest duration (median time, 50 vs. 52 minutes; *p* = 0.017). Overall, 80.3% of patients were treated with hypothermia. The proportion of patients treated with hypothermia was significantly higher in the NMBA group (88.0% vs. 63.6%; *p* < 0.001). In the NMBAs group, 60.9% received rocuronium and 39.1% received vecuronium. Among the patients whose infusion methods were available, 91.5% received NMBAs via continuous infusion (Supplementary Table 2). Midazolam was the most used sedatives (68.7%) and propofol usage was significantly lower (31.0% vs. 45.9%; *p* < 0.001) in the NMBA group. Analgesics were more used in the NMBA group (90.7% vs. 84.2%; *p* = 0.018). In addition, the incidence rates of pneumonia (31.2% vs. 22.3%; *p* = 0.019), ventilator-assisted pneumonia (27.3% vs. 14.1%; *p* < 0.001), and atelectasis (13.8% vs. 6.9%; *p* = 0.011) were significantly higher in the NMBA group as well. No significant differences were observed in incidence rates of temperature instability events and the various outcomes (Table 2).

**Table 1:** Patient characteristics overall and according to NMBA use.

	**n**	**Overall** n = 660^a^	**No NMBA** n = 209^b^	**NMBA** n = 451^c^	**p-value**^d^
Age, years	660	59[48,67]	63[51,70]	58[47,66]	<0.001
Sex	660				0.019
Female		98 (14.8%)	41 (19.6%)	57 (12.6%)	
Male		562 (85.2%)	168 (80.4%)	394 (87.4%)	
Comorbidities					
Hypertension	646	221 (34.2%)	71 (34.6%)	150 (34.0%)	0.877
Diabetes mellitus	646	143 (22.1%)	43 (21.0%)	100 (22.7%)	0.628
Dyslipidemia	646	100 (15.5%)	33 (16.1%)	67 (15.2%)	0.767
Heart disease	646	160 (24.8%)	49 (23.9%)	111 (25.2%)	0.728
Cerebrovascular disease	646	48 (7.4%)	10 (4.9%)	38 (8.6%)	0.092
Chronic kidney disease	646	25 (3.9%)	11 (5.4%)	14 (3.2%)	0.179
Dementia	646	2 (0.3%)	2 (1.0%)	0 (0.0%)	0.100
Others	646	173 (26.8%)	58 (28.3%)	115 (26.1%)	0.544
Location of cardiac arrest	660				0.415
Home		227 (34.4%)	70 (33.5%)	157 (34.8%)	
Public place		126 (19.1%)	39 (18.7%)	87 (19.3%)	
Street		114 (17.3%)	33 (15.8%)	81 (18.0%)	
Ambulance e		76 (11.5%)	30 (14.4%)	46 (10.2%)	
Workplace		75 (11.4%)	20 (9.6%)	55 (12.2%)	
Others		42 (6.4%)	17 (8.1%)	25 (5.5%)	
Initial cardiac rhythm at the scene	656				0.699
Shockable rhythm		503 (76.7%)	158 (75.6%)	345 (77.2%)	
Pulseless electrical activity		122 (18.6%)	39 (18.7%)	83 (18.6%)	
Asystole		31 (4.7%)	12 (5.7%)	19 (4.3%)	
Witnessed cardiac arrest	658	545 (82.8%)	167 (79.9%)	378 (84.2%)	0.175
Bystander CPR	652	387 (59.4%)	127 (61.1%)	260 (58.6%)	0.545
Intermittent ROSC before hospital arrival	648	67 (10.3%)	21 (10.3%)	46 (10.4%)	0.979
Initial cardiac rhythm on hospital arrival	659				0.830
Shockable rhythm		387 (58.7%)	126 (60.3%)	261 (58.0%)	
Pulseless electrical activity		193 (29.3%)	58 (27.8%)	135 (30.0%)	
Asystole		79 (12.0%)	25 (12.0%)	54 (12.0%)	
Cardiac rhythm at ECMO initiation	658				0.811
Shockable rhythm		427 (64.9%)	132 (63.2%)	295 (65.7%)	
Pulseless electrical activity		185 (28.1%)	62 (29.7%)	123 (27.4%)	
Asystole		46 (7.0%)	15 (7.2%)	31 (6.9%)	
Estimated cardiac arrest duration, min f	642	51 (41, 60)	52 (43, 63)	50 (40, 60)	0.017
Intermittent ROSC after hospital arrival	656				0.747
Before ECMO pump on		115 (17.5%)	35 (16.8%)	80 (17.9%)	
After ECMO pump on		541 (82.5%)	173 (83.2%)	368 (82.1%)	
Cause of cardiac arrest	660				0.719
ACS		437 (66.2%)	140 (67.0%)	297 (65.9%)	
Arrhythmia		90 (13.6%)	29 (13.9%)	61 (13.5%)	
Myocarditis		12 (1.8%)	2 (1.0%)	10 (2.2%)	
Myopathy		42 (6.4%)	10 (4.8%)	32 (7.1%)	
Other cardiac		24 (3.6%)	10 (4.8%)	14 (3.1%)	
Other non-cardiac		14 (2.1%)	6 (2.9%)	8 (1.8%)	
PE		24 (3.6%)	7 (3.3%)	17 (3.8%)	
Unknown		17 (2.6%)	5 (2.4%)	12 (2.7%)	

NMBA, neuromuscular blocking agent; CPR, cardiopulmonary resuscitation; ECMO, extracorporeal membrane oxygenation; ROSC, return of spontaneous circulation; ACS, acute coronary syndrome; PE, pulmonary embolism.

a Data are reported as medians [interquartile range] or numbers (percentage).

b NMBAs were infused within 24 hours of intensive care unit admission.

c NMBAs were not infused within 24 hours of intensive care unit admission.

d Pearson's chi-square test, Fisher's exact test, or Mann–Whitney U test as appropriate.

e Patients in whom spontaneous circulation was confirmed at the first emergency medical system team evaluation and then developed cardiac arrest.

f Estimated cardiac arrest duration was defined as follows: for patients whose location of cardiac arrest was ambulance, the time from cardiac arrest to the establishment of ECMO; for patients whose location of cardiac arrest was other than ambulance, the time from calling an ambulance to the establishment of ECMO.

**Table 2:** Interventions, complications, and outcomes.

	**N**	**Overall** n = 660^a^	**No NMBA** n = 209^b^	**NMBA** n = 451^c^	**p-value**^d^
Emergency coronary angiography	659	623 (94.5%)	194 (92.8%)	429 (95.3%)	0.187
Percutaneous coronary intervention	656	372 (56.7%)	122 (58.7%)	250 (55.8%)	0.493
Intra-aortic balloon pumping	659	552 (83.8%)	176 (84.2%)	376 (83.6%)	0.832
Target temperature for TTM	660				<0.001
Hypothermia (≤35 °C)		530 (80.3%)	133 (63.6%)	397 (88.0%)	
Normothermia (≥35.5 °C)		130 (19.7%)	76 (36.4%)	54 (12.0%)	
Renal replacement therapy	647	134 (20.7%)	48 (23.8%)	86 (19.3%)	0.197
Sedatitives use within 24 hours after ICU admission	660	660 (100.0%)	209 (100.0%)	451 (100.0%)	
Propofol	660	236 (35.8%)	96 (45.9%)	140 (31.0%)	<0.001
Midazolam	659	453 (68.7%)	132 (63.5%)	321 (71.2%)	0.057
Dexmedetomidine	659	31 (4.7%)	18 (8.7%)	13 (2.9%)	0.002
Analgesics use within 24 hours after ICU admission	659	584 (88.6%)	176 (84.2%)	408 (90.7%)	0.018
Fentanyl	659	559 (84.8%)	165 (78.9%)	394 (87.6%)	0.005
Morphine	657	8 (1.2%)	7 (3.3%)	1 (0.2%)	0.002
Temperature instability events					
Temperature deviated ≥0.5 °C from targeted temperature	538	74 (13.8%)	16 (10.1%)	58 (15.3%)	0.107
≥0.5 °C compared with targeted temperature	74	39 (52.7%)	12 (75.0%)	27 (46.6%)	0.044
≤0.5 °C compared with targeted temperature	74	36 (48.6%)	7 (43.8%)	29 (50.0%)	0.658
Temperature deviations ≥1.0 °C compared with targeted temperature	632	72 (11.4%)	24 (12.2%)	48 (11.0%)	0.651
≥1.0 °C compared with targeted temperature	72	41 (56.9%)	15 (62.5%)	26 (54.2%)	0.501
≤1.0 °C compared with targeted temperature	72	34 (47.2%)	12 (50.0%)	22 (45.8%)	0.738
Temperature <32.0 °C	632	8 (1.3%)	3 (1.5%)	5 (1.1%)	0.707
Infectious complications					
Pneumonia e	648	184 (28.4%)	46 (22.3%)	138 (31.2%)	0.019
Ventilator-associated pneumonia f	649	150 (23.1%)	29 (14.1%)	121 (27.3%)	<0.001
Urinary tract infection	648	27 (4.2%)	5 (2.4%)	22 (5.0%)	0.130
Catheter-related blood stream infection	649	50 (7.7%)	14 (6.8%)	36 (8.1%)	0.538
Other infection	646	33 (5.1%)	9 (4.3%)	24 (5.5%)	0.547
Sepsis	656	106 (16.2%)	32 (15.5%)	74 (16.4%)	0.769
Septic shock	656	58 (8.8%)	16 (7.8%)	42 (9.3%)	0.656
Atelectasis g	645	75 (11.6%)	14 (6.9%)	61 (13.8%)	0.011
Acute kidney injury	648	293 (45.2%)	102 (50.5%)	191 (42.8%)	0.069
Outcomes					
Hospital death	660	304 (46.1%)	100 (47.8%)	204 (45.2%)	0.531
Favorable neurological outcome at hospital discharge	660	177 (26.8%)	59 (28.2%)	118 (26.2%)	0.577
Length of intensive care unit stay, days	654	11 (6.0, 16.0)	10 (6.0, 15.5)	11 (7.0, 16.0)	0.267
Length of intensive care unit stay among survivors, days	350	13 (10.0, 18.0)	13 (10.0, 18.0)	13 (10.0, 19.0)	0.876
Length of hospital stay, days	655	21 (8.0, 41.0)	20 (7.0, 41.0)	21 (8.5, 40.0)	0.577
Length of hospital stay among survivors, days	351	37 (25.0, 56.0)	37 (26.8, 57.0)	36 (23.5, 54.0)	0.333
Length of mechanical ventilation, days	648	9 (6.0, 15.0)	8 (5.0, 14.0)	10 (6.0, 15.5)	0.225
Length of mechanical ventilation among survivors, days	345	11 (7.0, 17.0)	11 (7.0, 17.0)	11 (7.8, 16.2)	0.906

NMBA, neuromuscular blocking agent; TTM, targeted temperature management; ECMO, extracorporeal membrane oxygenation.

a Data are reported as medians [interquartile range] or numbers (percentage).

b NMBAs were infused within 24 hours of intensive care unit admission.

c NMBAs were not infused within 24 hours of intensive care unit admission.

d Pearson's chi-square test, Fisher's exact test, or Mann–Whitney U test as appropriate.

e Pneumonia was defined as follows: infiltrates on chest radiography and detection of bacteria in sputum cultures and at least one of the following: body temperature >38.0 °C, white blood cell count >12,000 cells/mL, and purulent sputum.

f Ventilator-associated pneumonia was defined based on the treating physician’s judgement and medical records.

g Atelectasis was defined as new infiltrates on chest radiography during ECMO management.

### Propensity score analysis

The C-statistic for the propensity score model was 0.700. After applying sIPW, the covariates between the NMBA and no NMBA groups were well-balanced, as shown in Table 3.

**Table 3:** Patient characteristics before and after stabilized inverse probability weighting.

	**Unadjusted group N = 633**			**Stabilized IPW group**		
	**No NMBA n = 202**	**NMBA n = 431**	**SMD**	**No NMBA**	**NMBA**	**SMD**
Age, years	60 (14)	55 (13)	0.33	56 (15)	57 (14)	−0.03
Sex			0.20			0.01
Female	40 (20%)	54 (13%)		(15%)	(15%)	
Male	162 (80%)	377 (87%)		(85%)	(85%)	
Location of cardiac arrest			0.17			0.05
Home	68 (34%)	149 (35%)		(34%)	(35%)	
Public place	39 (19%)	86 (20%)		(18%)	(19%)	
Street	32 (16%)	76 (18%)		(19%)	(18%)	
Ambulance a	29 (14%)	45 (10%)		(10%)	(11%)	
Workplace	19 (9.4%)	52 (12%)		(12%)	(11%)	
Others	15 (7.4%)	23 (5.3%)		(6.2%)	(5.8%)	
Initial cardiac rhythm at the scene			0.10			0.02
Asystole	12 (5.9%)	17 (3.9%)		(5.1%)	(5.1%)	
PEA	39 (19%)	80 (19%)		(18%)	(19%)	
Shockable	151 (75%)	334 (77%)		(77%)	(76%)	
Witnessed cardiac arrest	162 (80%)	364 (84%)	0.11	(82%)	(82%)	0
Bystander CPR	124 (61%)	250 (58%)	0.07	(59%)	(59%)	0.01
Estimated cardiac arrest duration, min b	56 (25)	52 (20)	0.20	54 (19)	53 (21)	0.06
Target temperature for TTM			0.62			0
Hypothermia (≤35 °C)	129 (64%)	383 (89%)		(81%)	(81%)	
Normothermia (≥35.5 °C)	73 (36%)	48 (11%)		(19%)	(19%)	

NMBA, neuromuscular blocking agent; IPW, inverse probability weighting; PEA, pulseless electrical activity; CPR, cardiopulmonary resuscitation; TTM, targeted temperature management; SMD, standardized mean difference.

Data are reported as means (standard deviation) or numbers (percentage).

a Patients in whom spontaneous circulation was confirmed at the first emergency medical system team evaluation and then developed cardiac arrest.

b Estimated cardiac arrest duration was defined as follows: for patients whose location of cardiac arrest was ambulance, the time from cardiac arrest to the establishment of extracorporeal membrane oxygenation; for patients whose location of cardiac arrest was other than ambulance, the time from calling an ambulance to the establishment of extracorporeal membrane oxygenation.

### In-hospital death

Overall, 304 patients (46.1%) died during hospitalization. The in-hospital mortality of patients in the NMBA and no NMBA groups was 45.2% (204/451) and 47.8% (100/209), respectively. In the multivariable Cox shared frailty model, NMBA use was not significantly associated with in-hospital mortality (adjusted HR 0.88; 95% CI, 0.64–1.14; *p* = 0.32; Table 4). The results did not substantially change after sIPW (adjusted HR 0.89; 95% CI, 0.64–1.25; *p* = 0.50; Table 5).

**Table 4:** Multivariate Cox shared frailty model and multivariable logistic regression model with generalized estimating equations: the association between neuromuscular blocking agent use and outcomes.

**Variable**	**Hospital death**				**CPC score 1 or 2 at discharge**				**Pneumonia**			
	**Event rate**	**Adjusted HR**^a^	**95% CI**	**p-value**	**Event rate**	**Adjusted OR**^b^	**95% CI**	**p-value**	**Event Rate**	**Adjusted OR**^b^	**95% CI**	**p-value**
NMBA use												
No NMBA	99 / 201 (49%)	Ref.			56 / 202 (28%)	Ref.			43 / 199 (22%)	Ref.		
NMBA	198 / 427 (46%)	0.88	0.67, 1.14	0.325	110 / 431 (26%)	0.85	0.60, 1.22	0.382	133 / 422 (32%)	1.52	0.85, 2.71	0.157

NMBA, neuromuscular blocking agent; CPC, Cerebral Performance Category; HR, hazard ratio; CI, confidence interval; OR, odds ratio; Ref., reference.

Estimated cardiac arrest duration was defined as follows: for patients whose location of cardiac arrest was ambulance, the time from cardiac arrest to the establishment of extracorporeal membrane oxygenation; for patients whose location of cardiac arrest was other than ambulance, the time from calling an ambulance to the establishment of extracorporeal membrane oxygenation.

a The multivariate Cox shared frailty model was adjusted for age, sex, witnessed cardiac arrest, bystander cardiopulmonary resuscitation, initial rhythm, location of cardiac arrest, and estimated cardiac arrest duration.

b The logistic regression model with generalized estimating equations was adjusted for age, sex, witnessed cardiac arrest, bystander cardiopulmonary resuscitation, initial rhythm, location of cardiac arrest, and estimated cardiac arrest duration.

**Table 5:** Association between neuromuscular blocking agent use and outcomes after inverse probability weighting.

**Variable**	**Hospital death**				**CPC score 1 or 2 at discharge**				**Pneumonia**			
	**Event Rate**	**Adjusted HR**	**95% CI**	**p-value**	**Event Rate**	**Adjusted OR**	**95% CI**	**p-value**	**Event Rate**	**Adjusted OR**	**95% CI**	**p-value**
NMBA use												
No NMBA	(50%)	Ref.			(27%)	Ref.			(22%)	Ref.		
NMBA	(46%)	0.89	0.64, 1.25	0.502	(26%)	0.94	0.59, 1.48	0.774	(31%)	1.59	0.74, 3.41	0.235

NMBA, neuromuscular blocking agent; CPC, Cerebral Performance Category; HR, hazard ratio; CI, confidence interval; OR, odds ratio; Ref., reference.

Adjusted HR was based on a weighted Cox regression model; adjusted OR was based on a weighted logistic regression model.

### Neurological outcome at hospital discharge

Overall, favorable neurological outcome at discharge was achieved in 177 patients (26.8%). The proportion of patients who achieved a favorable neurological outcome was similar in the NMBA and no NMBA groups (26.2% and 28.2%, respectively; Table 2). In the multivariable GEE logistic regression model, NMBA use was not significantly associated with favorable neurological outcome before (adjusted OR 0.85; 95% CI, 0.60–1.22; *p* = 0.38; Table 4) nor after sIPW (adjusted OR 0.93; 95% CI, 0.59–1.45; *p* = 0.70; Table 5).

### Pneumonia during hospitalization

Incidence of pneumonia during hospitalization was 31.2% in the NMBA group and 22.3% in the no NMBA group (Table 2). In the multivariable GEE logistic regression model, NMBA use and pneumonia were not significantly associated before (adjusted OR 1.52; 95% CI, 0.85–2.71; *p* = 0.16; Table 4), nor after sIPW (adjusted OR 1.53; 95% CI, 0.80–2.93; *p* = 0.20; Table 5).

### Subgroup analyses

In the subgroup analyses, no significant interactions were found between NMBA use and age, target temperature, or estimated cardiac arrest duration (Supplementary Table 3).

## Discussion

### Summary of the results

In this retrospective cohort study of OHCA patients who received ECPR and were treated with TTM, we evaluated the association between NMBA use and in-hospital mortality, neurological outcome, and incidence of pneumonia during hospitalization. After adjusting for covariates using multivariable regression models and propensity score analysis, no significant association was found between NMBA use and these outcomes.

### Relationship to previous studies

Several studies have investigated the effects of NMBAs in patients with PCAS treated with TTM.^23^, ^24^, ^25^, ^26^, ^27^, ^28^, ^29^ Although several observational studies reported an association between NMBA use and better outcomes,^25^, ^26^, ^28^, ^29^ our study found no such association. This disparity can be explained by several reasons.

The target population of this study differed from those in previous studies. Previous studies included few patients on ECMO, while ours only included those who underwent ECPR. NMBAs enable strict temperature management by preventing shivering. However, when ECMO is used, blood temperature can be directly regulated; therefore, patient body temperature can be controlled without the use of NMBAs. As a result, there may not be a significant association between NMBA use and patient outcomes. Indeed, the incidence of temperature-instability events in our study did not differ significantly between patients who received NMBAs and those who did not.

Several biases might have been present in previous studies which reported an association between NMBA use and favorable outcomes. First, patients who experience shivering, which is the main reason for administering NMBs, may have a lower degree of cardiac arrest-induced brain injury. The reasons are as follows. One study has reported that shivering is associated with favorable neurological outcomes in PCAS patients treated with hypothermia,^45^ suggesting that shivering is associated with less brain injury. In general, NMBAs are used to treat shivering; consequently, there is an association between the two. Therefore, it is possible that patients who received NMBAs had less brain injury than those who did not and consequently experienced a better outcome. This association is particularly strong when NMBAs are administered in a stepwise fashion according to the severity of shivering. In several previous studies which reported an association between NMBA use and good outcomes, they were administered in a stepwise protocol.^25^, ^28^ These studies might have been affected by such associations. To address this bias, we adjusted for several factors that influence brain injury related to cardiac arrest. Therefore, the extent of baseline brain injury owing to cardiac arrest seemed to be comparable in the NMBA and no NMBA groups.

Second, there was potential bias caused by patients who died or were subjected to a decision to limit treatment soon after ICU admission. It is common for PCAS patients to experience either of these^46^, ^47^ and such patients generally experience a poor outcome.^46^, ^47^ Furthermore, these patients may not have received an NMBA and were therefore classified in the no NMBA group, which would have resulted in an overestimation of the NMBA effect, as only patients who survived until NMBAs were administered were classified in the NMBA group. This immortality time bias is a common problem of observational studies.^48^ Several previous studies that reported an association between NMBAs and good outcomes did not mention death or the decision to limit treatment soon after ICU admission.^28^, ^29^ Therefore, immortality time bias might have been present. To address this bias, we excluded patients who died or received a DNAR order by the second calendar day following their admission. After addressing these biases, our findings are similar to those of previous randomized controlled trials.^23^, ^27^ Our findings might provide a more accurate estimate of the effects of NMBAs than previous observational studies.

Significant differences were observed in sedatives, with the NMBA group using less propofol. However, the impact of this for patient outcomes appear to be minimal because a study has reported no significant differences in outcomes between propofol and midazolam users among patients treated with ECPR.^49^

Pneumonia is a common complication of PCAS^50^ and is more likely in patients who receive NMBAs.^28^ Therefore, we also investigated the association between NMBA use and pneumonia. The incidence of pneumonia was significantly higher in patients treated with NMBAs in the crude analysis; however, after adjusting for covariates, no significant association was found. This is consistent with the findings of a previous study.^28^ NMBA use may contribute to pneumonia development by suppressing the cough reflex and impeding the elimination of airway secretions. Nevertheless, other factors such as severity of patient illness and differences in ICU management across hospitals may also play a role in pneumonia development. This might explain the lack of a significant association after adjusting for potential confounders.

### Significance and implications

Our study found no significant association between NMBA administration and favorable outcomes. While these results may discourage the routine use of NMBAs, they do not necessarily imply that NMBA administration should be avoided. Various physiological observations have suggested the potential adverse effects of shivering,^7^, ^8^, ^9^ which is effectively controlled by NMBAs. NMBAs may be a reasonable option for controlling shivering. We believe it is reasonable to evaluate the indications for NMBAs administration on a case-by-case basis.

### Strengths and limitations

To the best of our knowledge, this is the first study to evaluate the association between NMBA use and outcomes in OHCA patients who underwent ECPR and TTM. However, it had several limitations. First, data regarding shivering were not available. Therefore, it was not possible to determine whether NMBAs were administered to treat or prevent shivering. If NMBAs were administered to treat shivering, an association between NMBA use and less brain damage would have been established, as mentioned above. Consequently, we adjusted for several factors that influence brain damage, which should have attenuated this bias. Second, NMBA use was evaluated within the first 24 hours of ICU admission. The impact of the timing of NMBA use on outcomes beyond this period is unknown. However, this time frame is reasonable because shivering generally occurs during the TTM induction phase.^32^ Third, we did not investigate the presence of ARDS, for which NMBAs have been shown to be effective.^21^ Data regarding the presence of ARDS prior to cardiac arrest were not available; however, we believe that few patients had ARDS because most study patients had a cardiac cause for OHCA. Finally, the study was relatively small sample size and observational in design; therefore, these non-significant results might be due to being under-powered and causality could not be established. A randomized controlled trial with an adequate sample size would be required to confirm these findings.

## Conclusions

In this retrospective observational study of ECPR patients who received TTM, NMBA use within 24 hours of ICU admission was not significantly associated with patient outcomes. NMBA use should be based on individual clinical indications.

## Funding

This research did not receive any specific grant from funding agencies in the public, commercial, or not-for-profit sectors.

## Declaration of Generative AI and AI-assisted technologies in the writing process

During the preparation of this work the author used ChatGTP in order to improve readability of the text. After using this tool/service, the author reviewed and edited the content as needed and take(s) full responsibility for the content of the publication.

## CRediT authorship contribution statement

**Masatoshi Uchida:** Conceptualization, Methodology, Software, Investigation, Writing – original draft. **Migaku Kikuchi:** Conceptualization, Writing – review & editing. **Yasuo Haruyama:** Validation. **Toru Takiguchi:** Conceptualization, Writing – review & editing. **Toru Hifumi:** Conceptualization, Writing – review & editing. **Akihiko Inoue:** Conceptualization, Writing – review & editing. **Tetsuya Sakamoto:** Writing – review & editing. **Yasuhiro Kuroda:** Writing – review & editing.

## Declaration of Competing Interest

The authors declare that they have no known competing financial interests or personal relationships that could have appeared to influence the work reported in this paper.
