# Supplementary material for: Association between neuromuscular blocking agent use and outcomes among out-of-hospital cardiac arrest patients treated with extracorporeal cardiopulmonary resuscitation and target temperature management: A secondary analysis of the SAVE-J II study
Source: Resusc Plus. 2023 Sep 26;16:100476. doi: 10.1016/j.resplu.2023.100476 (PMC10540044; doi:10.1016/j.resplu.2023.100476)
Supplement: Supplementary data 1 [file mmc1.docx]

Supplemental file.

Table 1. Collected variables.

Patients' background;

| Variable name | Variable type | Variable values |
| --- | --- | --- |
| Age | Continuous | Years old |
| Sex | Nominal | Male, female, other |
| Hypertension | Dichotomous | Yes, No |
| Diabetes | Dichotomous | Yes, No |
| Dyslipidemia | Dichotomous | Yes, No |
| Heart disease | Dichotomous | Yes, No |
| Cerebrovascular disease | Dichotomous | Yes, No |
| Chronic kidney disease | Dichotomous | Yes, No |
| Dementia | Dichotomous | Yes, No |
| Other | Dichotomous | Yes, No |

OHCA characteristics;

| Location of cardiac arrest | Nominal | Home, public place, street, ambulance, workplace, others |
| --- | --- | --- |
| Initial cardiac rhythm at the scene | Nominal | Shockable rhythm, pulseless electrical activity, asystole |
| Witnessed cardiac arrest | Dichotomous | Yes, No |
| Bystander CPR | Dichotomous | Yes, No |
| ROSC before hospital arrival | Dichotomous | Yes, No |
| Cardiac rhythm at ECMO initiation | Nominal | Shockable rhythm, pulseless electrical activity, asystole |
| Estimated cardiac arrest duration | Continuous | Minutes |
| ROSC after hospital arrival | Dichotomous | Yes, No |
| Cause of cardiac arrest | Nominal | Acute coronary syndrome, arrhythmia, myocarditis, myopathy, other cardiac cause, pulmonary embolism, other non-cardiac cause, unknown |

Interventions;

| Emergency coronary angiography | Dichotomous | Yes, No |
| --- | --- | --- |
| Percutaneous coronary intervention | Dichotomous | Yes, No |
| Intra-aortic balloon pumping | Dichotomous | Yes, No |
| Target temperature at the beginning of TTM | Ordinal | 32.0, 32.5, 33.0, 33.5, 34.0, 34.5, 35.0, 35.5, 36.0, 36.5, 37.0, 37.5, 38.0 |
| Final target temperature after any adjustment | Ordinal | 32.0, 32.5, 33.0, 33.5, 34.0, 34.5, 35.0, 35.5, 36.0, 36.5, 37.0, 37.5, 38.0 |
| NMBs use at ICU admission | Dichotomous | Yes, No |
| NMBs use within 24 hours after ICU admission | Dichotomous | Yes, No |
| NMBs drugs | Nominal | Rocuronium, vecuronium, other |
| NMBs administrate route | Nominal | Bolus infusion, continuous infusion |
| NMBs doses | Continuous | Mg for bolus infusion, mg/hr for continuous infusion |
| Renal replacement therapy | Dichotomous | Yes, No |

Complications

| Temperature deviated ≥ 0.5 ℃ from targeted temperature | Dichotomous | Yes, No |
| --- | --- | --- |
| ≥ 0.5 ℃ compared with targeted temperature | Dichotomous | Yes, No |
| ≤ 0.5 ℃ compared with targeted temperature | Dichotomous | Yes, No |
| Temperature deviations ≥ 1.0 ℃ compared with targeted temperature | Dichotomous | Yes, No |
| ≥ 1.0 ℃ compared with targeted temperature | Dichotomous | Yes, No |
| ≤ 1.0 ℃ compared with targeted temperature | Dichotomous | Yes, No |
| Temperature < 32.0 ℃ | Dichotomous | Yes, No |
| Pneumonia | Dichotomous | Yes, No |
| Ventilator associated pneumonia | Dichotomous | Yes, No |
| Urinary tract infection | Dichotomous | Yes, No |
| Catheter related blood stream infection | Dichotomous | Yes, No |
| Other infection | Dichotomous | Yes, No |
| Sepsis | Dichotomous | Yes, No |
| Septic shock | Dichotomous | Yes, No |
| Atelectasis | Dichotomous | Yes, No |
| Acute kidney injury | Dichotomous | Yes, No |

Outcomes

| Hospital death | Dichotomous | Yes, No |
| --- | --- | --- |
| Favorable neurological outcome at hospital discharge | Dichotomous | Yes, No |
| Length of intensive care unit stay | Continuous | Days |
| Length of intensive care unit stay among survivors | Continuous | Days |
| Length of hospital stay | Continuous | Days |
| Length of hospital stay among survivors | Continuous | Days |
| Length of mechanical ventilation | Continuous | Days |
| Length of mechanical ventilation among survivors | Continuous | Days |

|  | n | n = 451*^1^* |
| --- | --- | --- |
| Neuromuscular blockade agents | 447 |  |
| Rocuronium |  | 272 (60.9%) |
| Vecuronium |  | 175 (39.1%) |
| Infusion methods*^2^* | 450 |  |
| continuous infusion |  | 365 (81.1%) |
| bolus infusion |  | 34 (7.6%) |
| unknown |  | 51 (11.3%) |
| NMBA, neuromuscular blocking agent | | |
| *1* Data are reported as median [interquartile range] for continuous variables and as N (percentage) for categorical variables. | | |
| *2* Continuous infusion; NMBA was infused continuously at ICU admission or within 24 hrs after ICU admission, bolus infusion; NMBA was only bolus infused, unkown; infusion methods were unavailable. | | |

Table 2. Details of neuromuscular blockade usage in patients treated with neuromuscular blockade agents

| Table 3. Subgroup analyses | | | | | | | |
| --- | --- | --- | --- | --- | --- | --- | --- |
| Subgroup | No NMBA |  | NMBA |  | Adjusted HR | 95% CI | P value for interaction |
| Age |  |  |  |  |  |  |  |
| ≥65 years old | 57/106 | (53.8%) | 66/127 | (51.2%) | 0.78 | 0.53, 1.16 | 0.583 |
| <65 years | 54/126 | (42.9%) | 139/316 | (44.0%) | 0.89 | 0.62, 1.29 |  |
| Target temperature for TTM |  |  |  |  |  |  |  |
| Hypothermia(≤35°C) | 63/128 | (49.2%) | 179/379 | (47.2%) | 0.89 | 0.67, 1.20 | 0.866 |
| Normothermia(>35°C) | 36/73 | (49.3%) | 19/48 | (39.6%) | 0.88 | 0.48, 1.61 |  |
| Estimated cardiac arrest duration |  |  |  |  |  |  |  |
| ≥50 minutes | 66/129 | (51.2%) | 110/209 | (52.7%) | 0.98 | 0.71, 1.37 | 0.466 |
| <50 minutes | 45/103 | (43.7%) | 95/234 | (40.6%) | 0.82 | 0.55, 1.24 |  |
| NMBA, neuromuscular blocking agent; ECPR, extracorporeal cardiopulmonary resuscitation; TTM, target temperature management | | | | | | | |
| The multivariate Cox shared frailty model was adjusted for age, sex, witnessed cardiac arrest, bystander CPR, initial rhythm, location of cardiac arrest, and estimated cardiac arrest duration. | | | | | | | |
| Estimated cardiac arrest duration was defined as follows: for patients whose location of cardiac arrest was ambulance, the time from cardiac arrest to the establishment of extracorporeal membrane oxygenation; for patients whose location of cardiac arrest was other than ambulance, the time from calling an ambulance to the establishment of extracorporeal membrane oxygenation. | | | | | | | |
